# Constraint-Induced Movement Therapy With or Without Neuromuscular Electrical Stimulation Versus Conventional Therapy for Upper Extremity Function in Chronic Stroke Survivors: Protocol for a Cluster Randomized Controlled Trial

**DOI:** 10.2196/68340

**Published:** 2025-08-28

**Authors:** Mahmoud M Dboba, Nor Azlin Mohd Nordin, Haidzir Manaf, Hanif Farhan Mohd Rasdi, Aznida Firzah Abdul Aziz

**Affiliations:** 1 Physiotherapy Program, Centre for Rehabilitation and Special Needs Studies, Faculty of Health Sciences, Universiti Kebangsaan Malaysia, Jalan Raja Muda Abdul Aziz Kuala Lumpur Malaysia; 2 Department of Physiotherapy, Faculty of Medical Technology, University of Tripoli Tripoli Libya; 3 Centre for Physiotherapy Studies, Faculty of Health Sciences, Universiti Teknologi MARA, Puncak Alam Campus Puncak Alam, Selangor Malaysia; 4 Occupational Therapy Program, Center for Rehabilitation and Special Needs Studies, Faculty of Health Sciences, Universiti Kebangsaan Malaysia, Jalan Raja Muda Abdul Aziz Kuala Lumpur Malaysia; 5 Department of Family Medicine, Faculty of Medicine, Universiti Kebangsaan Malaysia Kuala Lumpur Malaysia

**Keywords:** stroke, upper extremity, constraint-induced movement therapy, neuromuscular electrical stimulation, Post-stroke, stroke rehabilitation

## Abstract

**Background:**

Motor impairment is a common consequence of stroke, significantly impacting patients’ daily functioning and quality of life. More than 80% of stroke survivors experience upper extremity (UE) motor impairments, which severely impact their ability to carry out daily living activities. Combining motor rehabilitation techniques, such as constraint-induced movement therapy (CIMT), with sensorimotor stimulation methods, such as neuromuscular electrical stimulation (NMES), may be beneficial in enhancing UE functional recovery after a stroke. This combined approach could activate a broader range of brain regions, improving motor control. However, evidence for the effectiveness of these interventions when combined is still lacking.

**Objective:**

In this randomized controlled trial, we aim to determine the effectiveness of CIMT combined with NMES, CIMT alone, and conventional therapy in rehabilitating UE function in chronic stroke survivors.

**Methods:**

We will compare 3 intervention groups, 2 experimental groups (CIMT combined with NMES group and CIMT group) and 1 control group (conventional therapy), in this assessor-blinded, cluster randomized controlled trial. A total of 42 participants will be recruited and randomly assigned to 1 of the 3 groups. Participants in the CIMT group will undergo 6 shaping practice tasks (50 repetitions for each task, totaling 300 repetitions per session). The combined CIMT/NMES group will perform tasks similar to those carried out by the CIMT-only group, and a biofeedback electrical stimulator for NMES will also be provided. The control group will receive conventional neurodevelopmental therapy. All groups will receive 1 session per day of treatment for 3 consecutive days a week for 4 weeks. A blinded assessor will measure the interventions’ outcomes using standardized tests, including UE motor function, motor impairment, perceived motor function, and upper limb self-efficacy. All data will be analyzed descriptively, presenting the means and SDs. We will conduct inferential tests using a 2-way mixed ANOVA to evaluate the effects of the interventions and compare them between and within groups.

**Results:**

Funding for this study was provided by the Research and Ethics Committee of the Universiti Kebangsaan Malaysia (the National University of Malaysia) starting in November 2023. Between January 2023 and May 2023, we developed the initial ethics proposal and recruited research staff. Research ethics applications were submitted to the Research and Ethics Committee of the Universiti Kebangsaan Malaysia from May 2023 to November 2023. We anticipate publishing the study results in September 2025.

**Conclusions:**

This trial will provide insights into the effectiveness of CIMT combined with NMES, CIMT alone, and conventional therapy for UE rehabilitation in stroke survivors. Findings will be published in a peer-reviewed journal, and any updates based on emerging evidence will be provided. Any changes to the study methods will be documented in the final publication.

**Trial Registration:**

Australian New Zealand Clinical Trials Registry ACTRN12624000564550; https://www.anzctr.org.au/Trial/Registration/TrialReview.aspx?id=387308

## Introduction

### Background

Stroke is one of the most debilitating brain disorders, often leading to death or severe functional impairment. The global burden of stroke is exceptionally high in low- and middle-income countries, where increased incidence, mortality, disability, and economic strain are prevalent [1]. Stroke is the second leading cause of death and disability worldwide [2], with over 116 million healthy years lost annually due to disabilities and fatalities associated with stroke [3]. Among stroke survivors, more than 80% experience motor impairments in the upper extremities (UEs) [4], significantly limiting their ability to engage in social and daily activities. While approximately two-thirds of patients who have experienced a stroke can walk independently 6 months after the stroke, nearly half remain unable to functionally use the affected arm despite receiving treatment [5].

UE impairment is a common challenge for stroke survivors, often leading to significant motor dysfunction and impacting daily living activities [6,7]. Improving UE function through targeted rehabilitation is essential for enabling stroke survivors to perform daily tasks independently [8,9]. Constraint-induced movement therapy (CIMT) is a highly effective intervention for enhancing UE function after stroke [10] and is considered one of the most essential rehabilitation approaches for stroke recovery [11]. Numerous systematic reviews provide strong evidence supporting CIMT’s use across acute, subacute, and chronic phases of stroke rehabilitation [12-15]. Consequently, CIMT is recommended as a core intervention in clinical guidelines worldwide, including in the United Kingdom [16], Australia [16], and Canada [17]. In addition, research indicates that CIMT can enhance functional performance compared to traditional rehabilitation and increase the use of the most affected UE [18].

Despite the prevalence of motor impairments following a stroke, neuromuscular electrical stimulation (NMES) interventions are designed to enhance the quality of life and motor function by contracting weak muscles in the affected extremity. This approach aims to address paresis, restore fractionated movements, improve coordination and joint control, and decrease abnormal movement patterns, such as reduced spasticity and abnormal muscle synergies [19]. Clinical evidence supports the therapeutic efficacy of NMES as a complementary approach to traditional physiotherapy. Specifically, NMES for finger, arm, and wrist extension has shown the most significant clinical and neuroprosthetic benefits. Research indicates that individuals with moderate impairments within 2 years after a stroke experience essential improvements in UE function from NMES, especially when the stimulation is synchronized with the individual’s movement intent [19,20].

Motor function recovery is a critical aspect of stroke rehabilitation. However, in the chronic stage of stroke, the pace of functional recovery often slows considerably. This slow recovery rate, combined with the inherent tendency of humans to rely on bilateral arm coordination for functional activities, may lead to the development of compensatory movement strategies that hinder optimal recovery of the affected UEs. Humans instinctively use both arms in a coordinated fashion to carry out daily tasks. Following a stroke, this natural inclination to rely on bilateral arm coordination, coupled with the slow recovery of the affected arm, may lead patients to develop compensatory movements. While enabling individuals to cope with their impairments, these strategies limit the potential for optimal recovery of the affected UE. As patients become increasingly reliant on compensatory movements, they may fail to engage the impaired limb effectively, leading to a plateau in functional improvement. Consequently, rehabilitation professionals must recognize and address these compensatory strategies to promote optimal recovery and prevent long-term functional limitations in chronic stroke survivors. To overcome the challenges posed by compensatory strategies and promote recovery in the chronic stage of stroke, a combined approach integrating motor rehabilitation techniques, such as CIMT, with sensorimotor stimulation methods, such as NMES, may be beneficial [21]. Combined therapeutic interventions demonstrate significant potential for activating diverse brain regions involved in motor control, enhancing recovery following stroke. The clinical implications of these findings suggest that implementing multimodal interventions is essential for optimizing motor recovery in chronic stroke survivors. This approach is substantiated by functional magnetic resonance imaging studies revealing neuroplastic changes associated with combined therapy modalities, demonstrating enhanced cortical reorganization when peripheral stimulation is paired with intensive motor training [21-23]. The neuroimaging evidence provides a neurophysiological basis for the efficacy of combined interventions, offering valuable insights into evidence-based rehabilitation protocols that may inform clinical practice guidelines and future research directions in UE rehabilitation after stroke.

### Objectives

Although CIMT is an established intervention for stroke recovery, limited research exists on the synergistic effects of combining it with NMES. No clinical studies have reported the effectiveness of CIMT combined with NMES in comparison to CIMT alone or conventional therapy for treating UE dysfunction in patients who have experienced a stroke. Therefore, we aim to combine CIMT with NMES to explore the effect of their combination on UE dysfunction in chronic stroke survivors and compare it to CIMT alone and conventional therapy.

## Methods

### Trial Design and Setting

This study is an assessor-blinded, cluster, randomized controlled trial (RCT) comparing 3 intervention groups: 2 experimental groups (CIMT combined with NMES and CIMT alone) and 1 control group (conventional therapy). The trial will be conducted across 3 National Stroke Association of Malaysia (NASAM) rehabilitation centers. NASAM, a primary referral center for stroke survivors in Malaysia, operates 8 centers nationwide, providing rehabilitation services, peer support, and information to stroke survivors.

**Figure 1:**
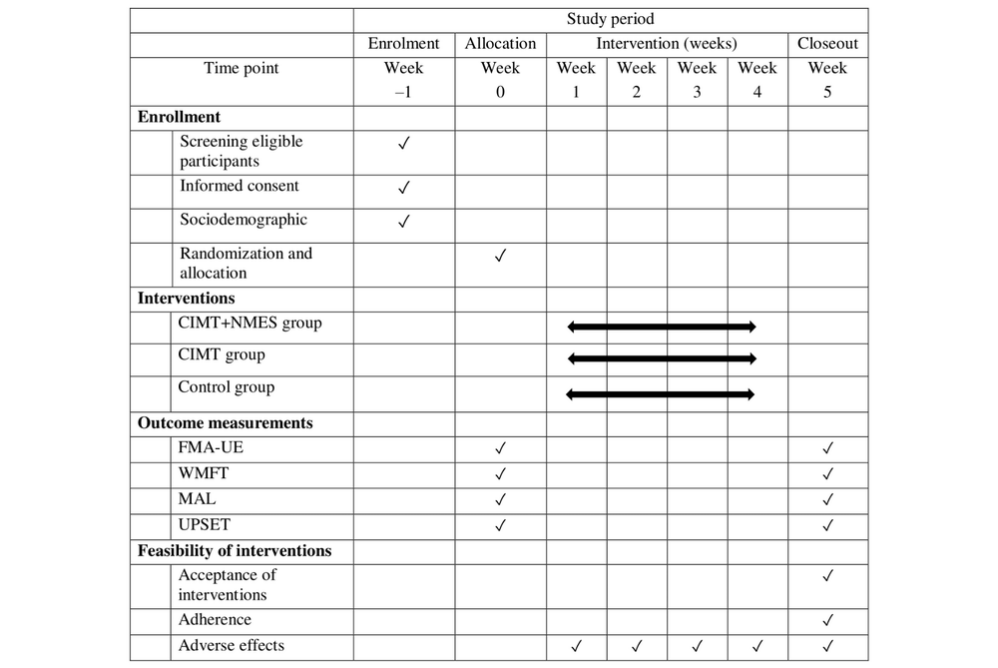
Flow diagram of the study. CIMT: constraint-induced movement therapy; FMA-UE: Fugl-Meyer Assessment for upper extremity; MAL: Motor Activity Log; NMES: neuromuscular electrical stimulation; UPSET: Upper Limb Self-Efficacy Test; WMFT: Wolf Motor Function Test.

**Figure 2:**
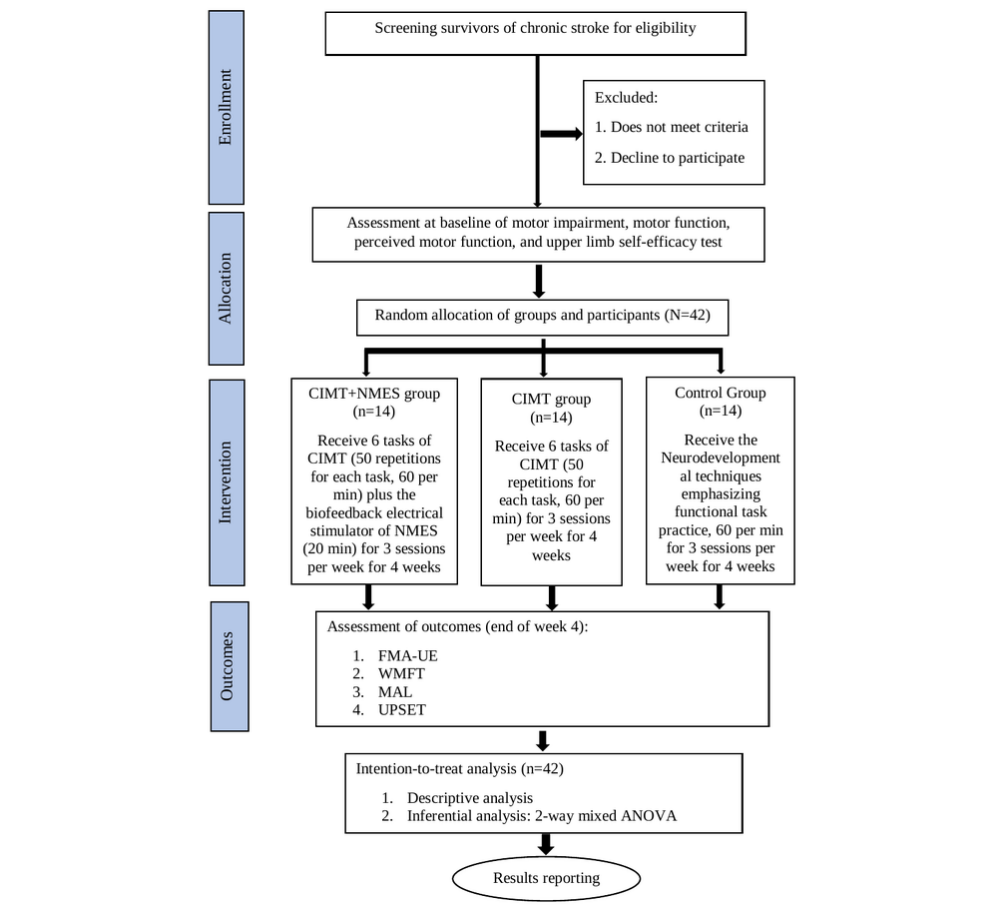
Flowchart of the study. CIMT: constraint-induced movement therapy; FMA-UE: Fugl-Meyer Assessment for upper extremity; MAL: Motor Activity Log; NMES: neuromuscular electrical stimulation; UPSET: Upper Limb Self-Efficacy Test; WMFT: Wolf Motor Function Test.

The study will be reported according to the CONSORT (Consolidated Standards of Reporting Trials) guidelines. The trial schedule is presented in Figure 1, while the trial design flowchart is provided in Figure 2. In addition, the study has been registered prospectively with the Australian New Zealand Clinical Trials Registry under registration number ACTRN12624000564550.

### Study Participants

The principal researcher will recruit eligible participants using the predefined inclusion and exclusion criteria, as described in Textbox 1 [24-28].

**Inclusion and exclusion criteria.**

**Inclusion criteria**

- Adults aged 18 to 80 years, with a diagnosis of ischemic or hemorrhagic stroke in the chronic phase (≥6 months after stroke)
- A score of ≥3 on the upper arm item of the Motor Assessment Scale
- A score of ≥44 on the Fugl-Meyer Assessment for upper extremity, with ≥10° active wrist extension and ≥10° active extension of thumb and additional digits
- A score of ≥26 on the Montreal Cognitive Assessment [24]. The patients must not have severe impairments in cognitive function, as they should understand the instructions and communicate
- A grade of ≤2 on the Modified Ashworth Scale for the upper extremity and lower extremity [25]
- No history of upper extremity injuries. History of upper injuries, such as a fracture or any deformity that can prevent patients from practicing massed tasks with the affected limb

**Exclusion criteria**

- Sensory loss score of ≥2 on the National Institutes of Health Stroke Scale sensory item. Such impairments can affect the ability to use the upper extremity in everyday activities [26]
- Patients with pacemakers or tumors owing to their contraindication to the implementation of the neuromuscular electrical stimulation [27,28]

### Recruitment and Randomization

Participants will be recruited from the NASAM. Three NASAM centers will be randomly assigned to 1 of 3 groups: the CIMT combined with the NMES group, the CIMT-only group, or the control group. Due to the specific nature of the therapies and the distinct characteristics of the centers, randomizing individual participants within centers is impractical. A cluster randomization approach will be used to minimize contamination among patients attending the same center. In addition, to minimize the risk of contamination between intervention groups, a cluster randomization approach will be used, wherein health care centers will serve as the unit of randomization. Each participating center will be randomly allocated to 1 of the 3 intervention arms using a computer-generated sequence administered by research personnel not involved in intervention delivery or outcome assessment. This concealed allocation process ensures allocation concealment and methodological rigor. Following randomization, all eligible patients who have experienced a stroke attending a given center will receive only the intervention protocol assigned to that facility. This methodological approach enables consistent intervention delivery within each site while substantially reducing the likelihood of cross-contamination between intervention groups—a critical consideration when evaluating health care provider–delivered rehabilitation protocols with behavioral components.

Eligible participants will provide written informed consent before their involvement in the study. The principal investigator or a trained research staff member not involved in therapy delivery will be responsible for obtaining consent from each participant. This process will take place only after participants have received a comprehensive explanation of the study procedures, ensuring that they clearly understand their involvement and the associated risks and benefits.

Following the informed consent process, baseline demographic information, including the participants’ names, ages, and gender, will be collected. Once demographic data have been obtained, the remaining baseline assessments will be conducted. Throughout the study, the confidentiality of all participants’ personal information and data will be strictly maintained in accordance with established research ethics guidelines and data protection regulations.

### Blinding

Outcome assessors will be blinded to the group assignments to ensure an unbiased evaluation of the results. However, given the significant differences in the therapies and the nature of the interventions, it is not feasible to blind the therapists or the patients involved in the study. Clinical staff must be aware of the specific therapy they are administering. Therapists will be informed that a study is being conducted on different modalities in other NASAM centers, but no particular details about the treatment regimens or locations will be provided. They will be aware of the treatment regime at their center but are reminded not to disclose this information to other therapists, either within or at different centers.

### Therapist Training and Standardization

Recognizing that therapist-related variability represents a significant potential source of bias in rehabilitation trials, we have developed comprehensive standardization procedures to minimize therapist-level confounding. While the complete elimination of therapist effects remains impossible in hands-on interventions, our multifaceted approach ensures maximum consistency across sites and health care providers.

All participating therapists must complete a 3-day training program before commencing study treatments. This standardized curriculum encompasses theoretical foundations underlying each intervention approach, extensive hands-on skill development with practice participants, and a detailed review of all protocol procedures. Competency assessment requires therapists to achieve a minimum score of 90% on written examinations covering theoretical knowledge and protocol specifications and satisfactory performance on practical skills assessments evaluated by master trainers. Weekly refresher sessions throughout the trial will maintain intervention quality, address emerging questions, and ensure continued protocol adherence.

To enhance baseline comparability across sites, participation is restricted to therapists possessing at least 5 years of stroke-specific rehabilitation experience. This requirement ensures foundational clinical expertise while creating greater homogeneity in health care provider characteristics. Each therapist will exclusively deliver 1 intervention type throughout the study, eliminating within-center variability and reducing contamination risk between treatment arms. The same therapist will treat all participants within their assigned center, maintaining consistency in intervention delivery style and therapeutic relationship development.

Structured peer observations using standardized feedback forms will maintain consistent intervention quality across sites. Detailed session plans specifying exercise sequences, progression criteria, and acceptable modifications will limit unwanted variability while preserving necessary clinical flexibility for individual participant needs. This comprehensive standardization approach minimizes therapist-related variability while enabling appropriate statistical control for unavoidable health care provider effects, strengthening our trial’s internal validity and interpretability.

### Interventions

The interventions in this study will take place on 3 consecutive days each week for 4 weeks. The study flowchart (Figure 2) provides a detailed overview of participant flow through the clinical trial.

#### CIMT Group

In the CIMT group, the shaping practice of CIMT will perform the following: picking up a cup from the table, taking it to the mouth, and putting it back; writing letters or drawing a circle; picking up an object (a pen or pencil) and transferring from right to left on the table; taking the hand from the lap to the head and sliding it from the front to the back; taking the hand to the nose; and putting and removing shoes. These 6 tasks will be performed 50 times, totaling 300 repetitions per session. The tasks were developed following a protocol outlined in a previous study [29].

Participants will complete hour-long CIMT sessions 3 times per week for 4 weeks, aiming to perform the 300 task repetitions within each session. This repetition target is supported by evidence from previous studies [30-32], showing that ≥300 daily repetitions can drive motor function recovery and can be achieved within 1 hour of focused practice. Structured progression criteria will adjust the tasks throughout the intervention to standardize the repetition counts while accommodating individual capacities.

Participants in the CIMT group will wear a padded or oven mitt on their unaffected UE for 90% of waking hours, 3 days per week, over the 4-week intervention period. On the basis of studies by Abdullahi [29], Wolf et al [33], and da Silva et al [34], this constraint protocol aims to promote intensive use of the affected limb during daily activities.

The selection of mitts was based on affordability, accessibility, and ease of maintenance considerations. Adherence to the constraint protocol will be monitored through participant self-report logs, verified by caregivers when possible, and direct therapist observations during treatment sessions.

Table 1 outlines the CIMT intervention framework, detailing the types of tasks, repetitions, sessions per day, and duration of task practice.

**Table 1:** Characteristics of the constraint-induced movement therapy intervention.

Task	Task repetitions per session, n	Sessions per day, n	Days per week, n	Weeks, n
Picking up a cup from the table, taking it to the mouth, and putting it back	50	1	3	4
Writing letters or drawing a circle	50	1	3	4
Picking up an object (pen or pencil) and transferring it from right to left on the table	50	1	3	4
Take the hand from the lap to the head and slide it from the front to the back	50	1	3	4
Taking the hand to the nose	50	1	3	4
Putting on and removing shoes	50	1	3	4

#### CIMT and NMES Group

Participants in the CIMT+NMES group will perform functional tasks similar to those in the CIMT group, with the addition of NMES delivered via a biofeedback electrical stimulator. During NMES sessions, participants will be seated with their paretic arm resting at their side and forearm pronated. Two stimulating electrodes will be placed over the extensor carpi radialis muscle belly. Participants will be instructed to relax the paretic arm while stimulation intensity is adjusted to elicit a sustained muscle contraction, known as a tetanic contraction, producing 20° to 25° of wrist extension without pain. NMES will be administered for 20 minutes per day, 3 days per week, over the 4-week intervention period.

NMES will be applied immediately following the completion of each CIMT session. This sequencing optimizes neuromodulatory effects while preserving volitional movement patterns during functional tasks [35,36]. By delivering NMES after the CIMT protocol, this approach allows for muscle activation without the influence of fatigue, potentially enhancing the therapeutic benefits of the combined intervention.

The NMES intervention will incorporate the use of an NMES stimulator (Balego EMS Digital Neuromuscular NMES Stimulator OTC) to deliver symmetric biphasic pulses. The stimulator will be set at a 35- to 50-Hz frequency with a 200- to 250-microsecond pulse width. Intensity will be individually adjusted to produce visible muscle contraction without discomfort, and electrode placement will target the wrist and finger extensor muscles.

#### Control Group

The control group will receive conventional rehabilitation therapy following a standardized protocol developed from established clinical guidelines [37,38]. This evidence-based approach ensures that participants in the control group receive consistent, high-quality care representing current best practices in stroke rehabilitation while maintaining a clear distinction from the experimental interventions.

Each 60-minute control session follows a structured format comprising 4 distinct therapeutic components. Sessions commence with 15 minutes of passive and active range of motion exercises systematically targeting all UE joints. Therapists guide participants through standardized movement sequences encompassing shoulder flexion, abduction, and rotation; elbow flexion and extension; and comprehensive wrist and finger movements. Each movement follows predetermined repetition counts with explicit progression criteria based on individual functional capacity.

The second component consists of 20 minutes of neurodevelopmental facilitation techniques. This segment incorporates weight-bearing activities performed in specified positions for predetermined durations, bilateral integration tasks systematically selected from our standardized task bank, and tone normalization techniques following established therapeutic principles. Task selection follows a structured decision algorithm that ensures consistency across therapists while permitting appropriate clinical judgment for individual participant needs.

Functional task practice occupies the subsequent 20 minutes, emphasizing activities of daily living relevant to participants’ rehabilitation goals. Task difficulty is systematically graded according to the Motor Assessment Scale scores, with compensatory strategy training incorporated when standardized criteria indicate clinical necessity. Each session concludes with 5 minutes of fine motor activities, including standardized pegboard tasks and grip strength exercises using specified resistance parameters.

All participating centers will receive identical therapy kits containing standardized materials to eliminate equipment-related variability. We have developed a comprehensive therapy manual featuring detailed photographic demonstrations, step-by-step instructions, and decision algorithms guiding exercise selection and progression. We will systematically review individual participant progress in weekly case discussions and ensure protocol adherence while maintaining necessary clinical flexibility within our structured therapeutic framework.

The principal researcher and health care professionals will administer therapy sessions in all groups. To ensure rigorous data collection and participant safety, all participants will have standardized logbooks to document their therapy sessions. The research team will monitor each participant’s adherence to the assigned intervention and track any potential adverse events, such as muscle cramps or soreness, every week.

For participants with cognitive or motor limitations that affect their ability to maintain the logbooks independently, assistance will be provided by caregivers or trained research assistants. All instances of such assistance will be carefully recorded to preserve data integrity and allow for appropriate analysis and interpretation of the results.

This multimodal monitoring approach, incorporating both self-report and direct observation, is designed to comprehensively assess adherence while accommodating the potential functional limitations of the stroke survivor population. By systematically tracking adherence and adverse events, the study aims to ensure participant safety, maximize data quality, and ultimately enhance the validity and generalizability of the findings.

Table 2 outlines the framework of all study interventions, including a comparison between the 3 groups.

**Table 2:** Framework of interventions: comparison between groups.

Characteristics	CIMT^a^ group	CIMT+NMES^b^ group	Control group
Total duration (min)	60	CIMT: 60; NMES: 20	60
Delivery	Researchers and health care professionals	Researchers and health care professionals	Researchers and health care professionals
Content	6 tasks of CIMT (50 repetitions for each task)	6 tasks of CIMT (50 repetitions for each task) + biofeedback electrical stimulator	Neurodevelopmental techniques emphasizing the functional task practice
Location	1 NASAM^c^ center	1 NASAM center	1 NASAM center
Frequency	1 session per day for 3 consecutive days a week for 4 weeks	1 session per day for 3 consecutive days a week for 4 weeks	1 session per day for 3 consecutive days a week for 4 weeks

^a^CIMT: constraint-induced movement therapy.

^b^NMES: neuromuscular electrical stimulation.

^c^NASAM: National Stroke Association of Malaysia.

#### Intervention Fidelity Monitoring

To ensure consistent intervention delivery across all sites and maintain protocol adherence, we have developed a comprehensive fidelity monitoring system based on the National Institutes of Health Behavior Change Consortium recommendations [39]. This multicomponent framework integrates standardized documentation, independent quality assurance, and technology-enhanced monitoring to maintain treatment integrity throughout the trial.

Our monitoring protocol incorporates standardized treatment fidelity checklists tailored explicitly to each intervention arm. These checklists document critical elements for every session, including exercise parameters, constraint application procedures, and NMES settings. Therapists will complete session-specific adherence forms immediately following each treatment, recording quantitative data on task repetitions completed, constraint wear time achieved, and stimulation parameters used. In addition, therapists will complete weekly self-assessment rubrics designed to promote reflective practice and facilitate early identification of protocol deviations.

Independent quality assurance measures will strengthen monitoring objectivity. The research coordinator will conduct biweekly observations across all sites using structured evaluation forms to assess protocol adherence systematically. An independent assessor uninvolved in the treatment delivery will randomly audit 20% of all sessions to ensure unbiased evaluation. With participant consent, we will video-record one session weekly per therapist for comprehensive offline analysis, enabling a detailed review of intervention technique and adherence verification.

Technology integration will enhance monitoring precision through automated data collection. NMES devices will generate electronic logs that objectively track all stimulation parameters, providing verifiable records of treatment delivery. We will systematically cross-validate participant-maintained logbooks with therapist records to verify reported constraint wear time and home practice completion. Weekly research team meetings will review aggregated adherence data, allowing prompt identification and resolution of any protocol deviations. This integrated monitoring approach balances rigorous quality control with practical feasibility, combining self-report measures, direct observation, and objective technological data to maintain optimal treatment fidelity throughout the trial.

### Outcome Measurements

#### Feasibility

The feasibility of all interventions will be assessed based on the measures described in subsequent sections.

#### Patient Acceptance

Stroke survivors’ perceptions of the therapy will be evaluated through a posttrial question asking, “What do you think about this therapy?” Responses will be categorized as positive or negative and analyzed descriptively.

#### Adherence Rate

Adherence will be measured as the percentage of prescribed therapy sessions completed (out of 12). Data will be collected from participants’ logbooks.

#### Adverse Effects

Exercise-related adverse effects, such as strain, cramps, and muscle soreness, will be monitored weekly and evaluated in posttrial assessments.

#### Repetition Adherence

The number of task repetitions will be calculated. If a participant is unable to complete all repetitions, the number of completed repetitions will be recorded, and the percentage of task completion will be calculated for adherence assessment. Therapists will adapt task complexity to maintain appropriate challenges while enabling maximum quality repetitions within individual capacity. This ensures standardized assessment while accounting for individual variability.

#### Constraint Adherence

Adherence to the constraint protocol will be monitored using daily logbooks for self-reported mitt wear, caregiver confirmation when available, and weekly therapist check-ins. The percentage of target constraint hours achieved will be calculated as a feasibility outcome. This multimodal approach ensures a comprehensive assessment of adherence, informing the interpretation of study outcomes and guiding future CIMT intervention design.

#### Intended Outcome Measures

##### Overview

Table 3 illustrates the framework for all study outcomes, including the instruments used to assess each variable. The details of the intended outcome measures for the interventions in the main study (RCT) are provided in subsequent sections.

**Table 3:** Framework of the study’s outcome measures and instruments.

Variables	Instruments
Motor impairment	Fugl-Meyer Assessment for upper extremity
Motor function	Wolf Motor Function Test
Perceived motor function	Motor Activity Log
Upper limb self-efficacy	Upper Limb Self-Efficacy Test

##### Motor Function Subscale of the Fugl-Meyer Assessment for Upper Extremity

The Fugl-Meyer Assessment (FMA-UE) will be used to assess motor function, coordination, and speed of the affected extremity, hand, and wrist after stroke. The FMA-UE is a well-established and reliable measure with excellent internal consistency [40,41]. In addition, it has been reported as a reliable and valid measure of poststroke upper limb motor impairment [42]. It assigns scores from 0 to 2 (0=cannot perform, 1=performs partially, 2=performs fully), with a total possible score of 66.

##### Wolf Motor Function Test

The Wolf Motor Function Test will assess the speed and accuracy of single- and multijoint movements and functional tasks of the UE. This test has been reported as a reliable and valid measure [43]. It includes 17 items, each rated on a scale from 0 to 5, where higher scores indicate greater ability (0=no ability and 5=normal ability, representing the ability to perform tasks with the same efficiency and coordination as an individual with no motor impairment).

##### Motor Activity Log

The Motor Activity Log is administered through a semistructured interview to assess how much and how effectively an individual uses their UE in their home environment. It comprises 2 subscales: the amount of use and the quality of movement, totaling 30 items that evaluate the use of the affected UE during daily tasks. Each item is scored on a 6-point Likert scale from 0 to 5, with higher scores reflecting greater perceived motor function. The scale has been reported to demonstrate both validity and reliability [44,45].

##### Upper Limb Self-Efficacy Test

The Upper Limb Self-Efficacy Test (UPSET) measures patients’ confidence in using their UEs for daily activities. It consists of 20 items rated on an 11-point Likert scale (0=no confidence and 10=high confidence). The UPSET has demonstrated good internal consistency and correlates well with other self-efficacy measures [46].

### Assessment of Outcomes

A blinded assessor unaware of group allocations will conduct baseline and postintervention assessments to prevent bias. The assessor will be trained in standardized assessment techniques and cannot access baseline data during postintervention evaluations.

A single trained assessor will perform all assessments at the respective rehabilitation centers while remaining blinded to group allocation and treatment throughout the study. This assessor will travel between centers to ensure consistent assessment procedures.

### Data Collection and Monitoring

All data will be meticulously recorded on case report forms and verified by the researcher. Raw data will be securely stored and accessible to researchers upon request, with appropriate measures to protect patient confidentiality. The principal researcher will be primarily responsible for ensuring the accuracy, integrity, and timeliness of the data entry process. To protect participant privacy, all files will be secured with passwords and only accessible to authorized research team members.

### Sample Size Calculation

We used the G*Power software (version 3.0.10; University of Düsseldorf) to calculate the required sample size. A mixed model ANOVA, a statistical method used to analyze the effects of interventions over time and between groups, was selected to analyze the interventions’ time, group, and interaction effects. The *F* test (ANOVA repeated measures within and between interactions), a specific type of statistical test used in the mixed model ANOVA, was selected for this analysis. With a study power of 80%, an α level of .05, and an effect size of 0.25 (based on the primary outcomes from a previous CIMT study [47]), a minimum sample size of 42 participants (14 in each group) is required. If a group has more than 14 eligible participants, the first 14 to provide consent will be recruited based on the referral date. This first-come, first-served approach ensures transparency in participant selection while maintaining cluster integrity.

An intention-to-treat analysis, which includes all randomized participants, regardless of their adherence to the protocol or completion of the study, will be implemented for this RCT. Under this approach, all recruited participants will be included in the final analysis regardless of protocol adherence, withdrawal, or discontinuation. For participants who withdraw or discontinue the treatment, their baseline data will be used as exit data in the primary analysis. This conservative approach acknowledges the potential impact of missing data while maintaining randomization integrity. Sensitivity analyses using multiple imputation techniques, which are statistical methods used to handle missing data, will be conducted to assess the robustness of findings against different missing data assumptions. This analytical strategy aligns with CONSORT guidelines for reporting randomized trials and addresses potential selection bias that might occur with complete-case analysis.

### Statistical Analysis

Data will be analyzed using IBM SPSS Statistics software (version 29.0), adhering to the intention-to-treat principle, which includes all randomized participants regardless of protocol adherence. Participant characteristics and baseline measures will be summarized using descriptive statistics, including mean, SD, median, and IQR.

Before primary analyses, data will be assessed for normality using the Shapiro-Wilk test and homogeneity of variance using the Levene test. For outcomes meeting parametric assumptions, a 2-way mixed model ANOVA will be used to compare the effects of the interventions’ interactions between and within groups. Appropriate transformations or nonparametric alternatives will be applied in cases of violated parametric assumptions. Potential confounding variables, such as baseline motor function and stroke severity, will be included as covariates to provide adjusted treatment effect estimates. Statistical significance will be set at *P*<.05 for all analyses.

The linear mixed-effects models that include the center as a fixed effect covariate and random intercepts at the participant level will be used to address residual center-specific effects without invoking a design effect. This modeling framework accommodates hierarchical data structures in single-cluster designs while preserving valid inference for group-by-time interactions.

Missing data will be addressed using multiple imputation techniques, with sensitivity analyses comparing results to those obtained using the baseline observation carried forward method. This comprehensive approach ensures a robust interpretation of intervention effects while preserving statistical power.

### Ethical Considerations

This study will be conducted in accordance with the Declaration of Helsinki and Good Clinical Practice guidelines. Ethics approval has been granted by the Research and Ethics Committee of the Universiti Kebangsaan Malaysia (RECUKM; the National University of Malaysia; study ID UKM PPI/111/8/JEP-2023-596). All participants will provide informed consent before participating, and their privacy will be kept confidential.

The study findings will be disseminated through publication in peer-reviewed journals and presentations at national and international congresses. A summary of the trial results will also be made available on the Australian New Zealand Clinical Trials Registry database. All necessary regulatory approvals for the study have been obtained. Upon trial completion, participants will receive personalized feedback regarding their involvement and the study outcomes.

## Results

Funding for this study from RECUKM began in November 2023. Between January 2023 and May 2023, we drafted the initial ethics proposal and hired the research staff. Research ethics applications were submitted to the RECUKM from May 2023 to November 2023. We anticipate publishing the study results in 2025.

This study will evaluate the effectiveness of CIMT combined with NMES, CIMT alone, and conventional neurodevelopmental therapy in rehabilitating UE function in chronic stroke survivors. The findings could enable physiotherapists to develop innovative therapies to promote UE function and assist in identifying the most effective treatments for poststroke UE dysfunction. We hypothesize that CIMT combined with NMES will be more effective than the other 2 therapies in improving UE function among survivors of stroke.

## Discussion

### Anticipated Findings

This study evaluates the effects of combining CIMT with NMES on UE function among chronic stroke survivors. We hypothesize that the combined CIMT+NMES intervention will produce superior improvements in motor function and perceived functional use compared to CIMT alone or conventional therapy. This dual approach leverages complementary mechanisms: CIMT addresses learned nonuse through intensive task practice [48], while the NMES facilitates muscle activation and enhances proprioceptive feedback to support voluntary movement.

We examined current rehabilitation paradigms for chronic stroke to contextualize our approach within existing evidence. CIMT has established efficacy for improving UE function in selected populations of individuals who have experienced a stroke through forced use and mass practice tasks [49]. Similarly, NMES demonstrates clear benefits for muscle strengthening and motor unit recruitment in paretic limbs [50]. However, a few studies have investigated their combined therapeutic potential [10]. Recent evidence indicates that multimodal rehabilitation approaches may amplify neuroplastic changes through synergistic cortical reorganization mechanisms. Functional magnetic resonance imaging studies have documented enhanced cortical activation patterns when peripheral electrical stimulation accompanies intensive motor training, suggesting accelerated neural adaptation compared to single interventions [21].

This study advances this emerging field by providing the first systematic comparison of combined CIMT+NMES therapy against established monotherapies. This investigation addresses a critical gap in stroke rehabilitation evidence, particularly for chronic stroke survivors who typically experience limited recovery with conventional approaches. By examining whether combined interventions overcome the ceiling effects often observed with single modalities, our findings will inform evidence-based treatment selection for this challenging population.

### Strengths and Limitations

This trial incorporates several methodological strengths that enhance scientific rigor and clinical applicability. Blinded outcome assessors evaluate all participants using validated instruments spanning the International Classification of Functioning, Disability, and Health framework, ensuring a comprehensive assessment of motor impairment, activity limitations, and participation restrictions. Our standardized intervention protocols, supported by detailed treatment manuals and competency-based therapist training, promote consistent implementation across sites. The pragmatic trial design, conducted within existing rehabilitation centers using available clinical resources, enhances external validity and facilitates future implementation.

We acknowledge specific limitations inherent in our study design. While pragmatically necessary given resource constraints, the single-cluster-per-arm randomization strategy prevents traditional design effect calculations in our sample size calculation. We have addressed this limitation through conservative effect size selection and comprehensive statistical modeling that includes the center as a fixed effect covariate. Our mixed ANOVA approach protects against type I error inflation while maintaining adequate sensitivity for detecting clinically meaningful differences. The absence of long-term follow-up assessments limits our understanding of treatment durability; however, immediate postintervention outcomes will establish the foundation for future longitudinal investigations.

Despite rigorous standardization procedures, therapist-delivered interventions inevitably introduce some variability. We minimize this concern through mandatory competency assessments and structured fidelity monitoring. These mitigation strategies ensure that observed treatment differences reflect intervention efficacy rather than health care provider variability.

### Clinical Implications and Future Directions

Our findings will directly inform clinical decision-making regarding optimal rehabilitation strategies for chronic stroke survivors with persistent UE deficits. Should the combined intervention demonstrate superiority, clinicians will gain evidence supporting integrated treatment protocols that maximize functional recovery potential. The comprehensive outcome battery, including motor function (FMA-UE), activity performance (Wolf Motor Function Test), perceived use (Motor Activity Log), and self-efficacy (UPSET), provides multidimensional insights into treatment effects across the International Classification of Functioning, Disability, and Health domains.

Future research should expand upon our foundation through several critical avenues. Large-scale multicenter trials incorporating cluster randomization with appropriate design effects will establish definitive efficacy estimates. Extended follow-up periods of 6 to 12 months will determine whether initial gains translate into sustained functional improvements. Neurophysiological assessments, including transcranial magnetic stimulation and electroencephalography, could elucidate the cortical mechanisms underlying potential synergistic effects [51].

### Dissemination Plan

We have developed a comprehensive dissemination strategy to ensure rapid translation of findings into clinical practice. Primary dissemination will occur through publication in high-impact rehabilitation journals with open-access options to maximize accessibility. Conference presentations at international forums will facilitate direct engagement with clinical and research communities.

To accelerate clinical implementation, we will develop practitioner-oriented resources, including evidence summaries, clinical decision algorithms, and video training modules demonstrating proper technique. Collaboration with professional organizations will support integration into clinical practice guidelines and continuing education curricula. Patient-centered knowledge translation activities include creating accessible educational materials explaining treatment options and expected outcomes, available in multiple languages to serve diverse populations. Partnerships with stroke survivor organizations will ensure broad distribution through established communication channels, empowering patients to advocate for evidence-based care.

### Conclusions

This protocol addresses a critical gap in stroke rehabilitation evidence through rigorous evaluation of combined therapeutic approaches, integrating CIMT with NMES to target both voluntary movement control and direct muscle activation through complementary neuroplastic mechanisms. By systematically examining whether multimodal interventions produce synergistic effects exceeding those of single treatments, our study advances rehabilitation science through a comprehensive methodological framework that incorporates standardized protocols, quantitative fidelity monitoring, and cluster-appropriate statistical modeling, ensuring reliable evidence generation for clinical practice guidelines. Beyond establishing comparative efficacy, this research will elucidate optimal dosing parameters and practical implementation strategies for complex rehabilitation interventions, enabling clinicians to make informed decisions about resource allocation and treatment selection for chronic stroke survivors while contributing essential evidence toward developing personalized, evidence-based rehabilitation strategies that maximize functional recovery in this underserved population.

## Appendix

Multimedia Appendix 1 SPIRIT (Standard Protocol Items: Recommendations for Interventional Trials) checklist.

## Abbreviations

CIMT: constraint-induced movement therapy
CONSORT: Consolidated Standards of Reporting Trials
FMA-UE: Fugl-Meyer Assessment for upper extremity
NASAM: National Stroke Association of Malaysia
NMES: neuromuscular electrical stimulation
RCT: randomized controlled trial
RECUKM: Research and Ethics Committee of the Universiti Kebangsaan Malaysia
UE: upper extremity
UPSET: Upper Limb Self-Efficacy Test

## Data Availability

The datasets generated and analyzed during this study are available from the corresponding author upon reasonable request.
